# Intubation Related Laryngeal Injuries in Pediatric Population

**DOI:** 10.3389/fped.2021.594832

**Published:** 2021-02-10

**Authors:** Karma Lambercy, Laurence Pincet, Kishore Sandu

**Affiliations:** Head and Neck Surgery Department, Centre Hospitalier Universitaire Vaudois, Lausanne, Switzerland

**Keywords:** acquired laryngeal stenosis, acquired subglottic stenosis, endotracheal intubation, tracheotomy, laser surgery, airway management

## Abstract

**Introduction:** Laryngeal intubation related lesions (LIRL) in pediatric patients cause extreme morbidity in both elective and emergency settings. It has a wide range of presentations from minor laryngeal edema to a life-threatening airway obstruction. We report here our units' experience with LIRL in neonates, infants, and small children.

**Material and Methods:** This is a retrospective monocentric cohort study between January 2013 and April 2019.

**Results:** Thirty-nine patients with intubation lesions were included in the study. We looked at the lesions type, characteristics, management, and outcome. Half the patients were premature and having comorbidities. Main LIRL were subglottic stenosis (31%), ulcers (26%), granulations (18%), retention cysts (18%), posterior glottic stenosis (13%), and vocal cords edema (5%). Unfavorable lesions causing airway stenosis were associated with an intubation duration of over 1 week and were an important factor in causing airway stenosis (*p* < 0.05). The endoscopic treatment performed for these lesions was lesion and anatomical site-specific. Tracheostomy was needed in five patients, and was avoided in another two. Seven patients (18%) received open surgery prior to their decannulation.

**Conclusions:** LIRL management is challenging and stressful in the pediatric population and optimal treatment could avoid extreme morbidity in them. Intubation duration and associated comorbidities are important factors in deciding the severity of these lesions. Protocols to prevent the formation of these lesions are critical.

## Introduction

Laryngeal injury because of endotracheal intubation in the pediatric population continues to contribute to patient morbidity, in both elective and emergency settings. It has a wide range of presentations from minor laryngeal edema that heals spontaneously to a life-threatening airway obstruction (1). Patients' co-morbidities (prematurity, cardiopulmonary defects) and hypoxia status play a role in the tissue healing process, which may increase the propensity for the development of chronic intubation-related laryngeal lesions (1, 2). Various risk factors contributing to the development of laryngeal lesions in children are: patient-factors (prematurity, cardiopulmonary comorbidities), intubation technique (in emergency, inexperienced team), endotracheal tube (large size, cuffed tube), longer duration of intubation, infection and inadequate patient sedation (3–7).

Endoscopic visualization of the larynx is crucial in assessing the intubation trauma in children, as the severity of symptoms may not always correlate with the degree of laryngeal injury present. Laryngeal endoscopy provides a more sensitive guide in visualizing the laryngeal sub-sites and characterizing the damage done in them (8).

Various classification systems (2) exist to describe severity of the LIRLs ranging from mild erythema and edema to severe granulation tissue formation, mucosal ulceration, and cartilage exposure. Schweiger et al. (9) classified the injuries as per affection of laryngeal sub-sites and were able to prognosticate their outcomes.

In this commentary, we retrospectively look at our units' experience in managing pediatric patients with intubation-related laryngeal injury. Following this, we propose a management protocol aimed at reducing morbidity related to intubation injuries.

## Materials and Methods

After an internal review board clearance, we retrospectively collected data on all pediatric patients (age under 18 years) who presented with laryngeal intubation related lesions (LIRL) between January 2013 and April 2019. Patients included either had the intubation done in the pediatric intensive care unit (PICU) or during operations at our center, and those with LIRL referred to our center from other hospitals. Emergency intubation setting was considered when patients primarily presented or developed respiratory distress in a hospital or an outside-hospital setting. Elective intubations were those done in the PICU or prior to a surgery. All intubations done in the PICU (even those previously extubated after a surgery) were marked as medical indications. Patients intubated in the operation rooms and who remained non-extubated were surgical indications. Information was retrieved from hospital treatment charts and appropriate consent from the parents or guardians was obtained.

Data analyzed included: age at the first endoscopy, sex, comorbidities, type of lesion(s), site(s), localization(s), symptom(s) suggesting the lesion, duration, and history of prior intubation(s), manner of intubation, and the treatment offered.

Incipient subglottic stenosis (iSGS) was defined as progressing cicatrical airway stenosis that failed 2 prior endoscopic treatments. SGS was classified according to the Cotton-Myer classification (3): grade I- lumen obstruction up to 50%, II- 51–70% obstruction, III- 71–99%, IV−100%. The posterior glottic stenosis was classified according to the Bogdasarian classification (10, 11): type 1- inter-arytenoid cicatricial band but intact posterior glottic mucosa, 2- dense scar in the posterior glottis, 3- unilateral cricoarytenoid joint (CAJ) ankylosis, 4- bilateral CAJ ankylosis.

Temporary tracheostomy (done within 3 months after intubation) or a single-stage airway expansion surgery was not considered failure of the primary management strategy. The management strategy was deemed failure if the patient got a long-term tracheostomy.

All patients underwent transnasal flexible laryngotracheobronchoscopy under spontaneous breathing to establish the laryngotracheal dynamics. Under general anesthesia, examination of the supra-glottis, glottis, subglottis and the trachea was done with rigid 0° and 30° telescopes (4.0/2.9 mm endoscopes Storz, Germany).

We divided the LIRL into favorable and unfavorable depending upon them evolving into critical airway stenosis. Favorable lesions (FL) were erythema, mild edema, and small non-obstructing subglottic retention cyst(s). Unfavorable lesions (UFL) were moderate-severe edema with mucosal ulceration, posterior glottic and circumferential subglottic granulations, obstructing subglottic retention cyst(s), severe bilateral arytenoids and circumferential cricoid cartilage exposure and severe cuff / tip of the tube lesions.

Treatment of UFL was performed under suspension microlaryngoscopy (SML) and successive apnea using a *small-for age* Portex blue line endotracheal tube. Routine cold microlaryngoscopy instruments were used to remove granulations, polyps and unhealthy cicatricial tissue. Bleeding was controlled using 1:100,000 adrenaline-soaked patties. CO_2_ laser (Ultra-pulse Duo, Lumenis) connected to a microscope (Leica, Germany) and a micromanipulator (160/400 mm, Coherent, USA) was used to marsupialise retention cysts with the following parameters: ultrapulse mode, 100–125 mJ/cm^2^, defocused beam and 250 microspot. Topical injection of triamcinolone acetonide (Kenacort^R^ 40 mg/ml Bristol-Myers Squibb S.r.l.) was made into thin circumferential stenosis and cicatricial bands prior to making Mercedes Benz shape mucosal incisions (at 12, 4, and 8 o'clock positions) using either, a fine Blitzer laryngeal knife (Integra^R^ ENT) or sharp focused CO2 laser beam. Subsequently, balloon dilation was performed using an age-appropriate size Cordis^R^ or CRE^R^ pulmonary balloons. Diprogenta^R^ ointment (Betamethasone-Gentamycin; MSD Merck, Sharp & Dohme AG) was applied to the damaged area at the end of the treatment.

Severe granulations were first excised using cold instruments and then topical Mitomicin C (1 mg/2 mL) on a cotton patty was applied for 2 min.

Extubated patients were put on CPAP and closely monitored in the PICU. The non-invasive ventilation was progressively weaned off.

In case if re-intubation was necessary, one size smaller tube was used to intubate, a liberal diprogenta ointment plug was placed in the larynx around the tube and extubation was again tried after 48 h.

In case of recurrent incipient stenosis, an open surgery was performed to avoid a tracheostomy (12). If the tracheotomy was necessary, it was done between the lower border of the cricoid and the first tracheal ring or between the 6th and 7th rings (3). An age-appropriate laryngeal stent (Monnier LT mold) (13) was inserted under SML to allow healing around the stent in laryngeal abduction. The mold was fixed endolaryngeally using Lichtenberger's needle holder and 3.0 Prolene suture (Ethicon, Johnson & Johnson). The mold was removed endoscopically under SML after 2–3 weeks.

## Results

We included 39 patients with age ranging from 1 day to seventeen years (mean age = 3.35 years), male/female ratio of 1.17 and having a minimum follow up period of 12 months. The referring clinicians were contacted by e-mailing for patients from other institutions. Majority of patients were under 2 years (28 patients, 72%) and almost half of these were premature (19 patients, 49%). There were 6 patients with cardiac defects and 3 had genetic abnormalities (Table 1). Laryngeal lesions that needed intubation were bilateral vocal cord palsy (*n* = 3), glottic web with 60% obstruction and laryngeal edema (*n* = 1) and severe laryngitis having edema (*n* = 3).

**Table 1:** Patients' and lesions' characteristics.

**Characteristics**	**Prevalence**
**AGE**	
- Mean - Median Subcategories: ■ ≤ 1 year ■ > 1 year to ≤ 2 years ■ > 2 years	1 days to 17 years old • 1,224 days (3,4 y) • 547 days (1,5 y) ■ 17 (44%) ■ 11 (28%) ■ 11 (28%)
**SEX**	
Male/Female ratio - Male - Female	1,17/1 21 18
**COMORBIDITIES**	
- Prematurity - Laryngeal pathology: ° Congenital vocal cord palsy ° Laryngeal web ° Laryngitis - Cardiac malformations: ° Fallot tetralogy ° Patent arterial duct ° Other - Genetic abnormality: ° Down syndrome ° Turner syndrome ° Heterozygote 15q26 deletion - Other: ° Brain tumor ° Esophageal atresia ° Cerebral palsy ° Necrotizing pneumonia ° Pulmonary atresia ° Mental handicap ° Necrotizing enterocolitis	19 (49%) 7 (18%) 3 1 3 6 (15%) 1 4 1 3 (8%) 1 1 1 2 1 1 1 1 1 1
**INTUBATION MODE**	
- In emergency - Elective	33 (85%) 6 (15%)
**INTUBATION REASON**	
- Medical - Surgical	30 (77%) 9 (23%)
**REPEATED INTUBATIONS**	
- 1 intubation - Unique intubation	18 (46%) 21 (54%)
**DURATION OF INTUBATION (DAYS)**	
Average: - Short (1 ≤ week) - Long (>1 week to ≤1 month) - Prolonged (> 1 month)	23, CI 95% [15.4–30.8] 19 (49%) 8 (20%) 12 (31%)
**DIAGNOSIS**	
- Granulation - Sub-glottis stenosis - Polyps - Posterior glottis stenosis - Cyst - Arytenoid ulcer - Edema	7 (18%) 12 (31%) 1 (3%) 5 (13%) 7 (18%) 10 (26%) 5 (13%)
**STENOSING EFFECT**	
- Not stenosing - Stenosing	15 (38%) 24 (62%)
**LEVEL OF LESION**	
- Supra-glottis - Glottis - Sub-glottis	1 (2,5%) 17 (44%) 27 (69%)
**LOCALIZATION**	
- Anterior - Posterior - Anteroposterior	6 (15%) 21 (54%) 12 (31%)
**SIDE**	
- Unilateral - Bilateral	8 (21%) 31 (79%)
**PRESENTATION**	
- Dyspnea - Stridor - Dysphonia - Extubation failure - Tracheotomy - Other: (aphonia, asymptomatic)	15 (38%) 20 (51%) 7 (18%) 5 (13%) 5 (13%) 2 (5%)
**APPEARANCE OF SYMPTOMS**	
- Early (≤24 h) - Medium (>24 h to ≤ 1 month) - Delayed (> month)	19 (49%) 7 (18%) 13 (33%)

Thirty-three patients (84.6%) were intubated in emergency, 19 patients (49%) had repeated multiple intubations and 20 patients (51%) developed LIRL after one single intubation. All patients were intubated for more than 24 h, 19 patients (49%) were intubated for <1 week, and 12 patients (31%) remained intubated for more than a month.

**Figure 1:**
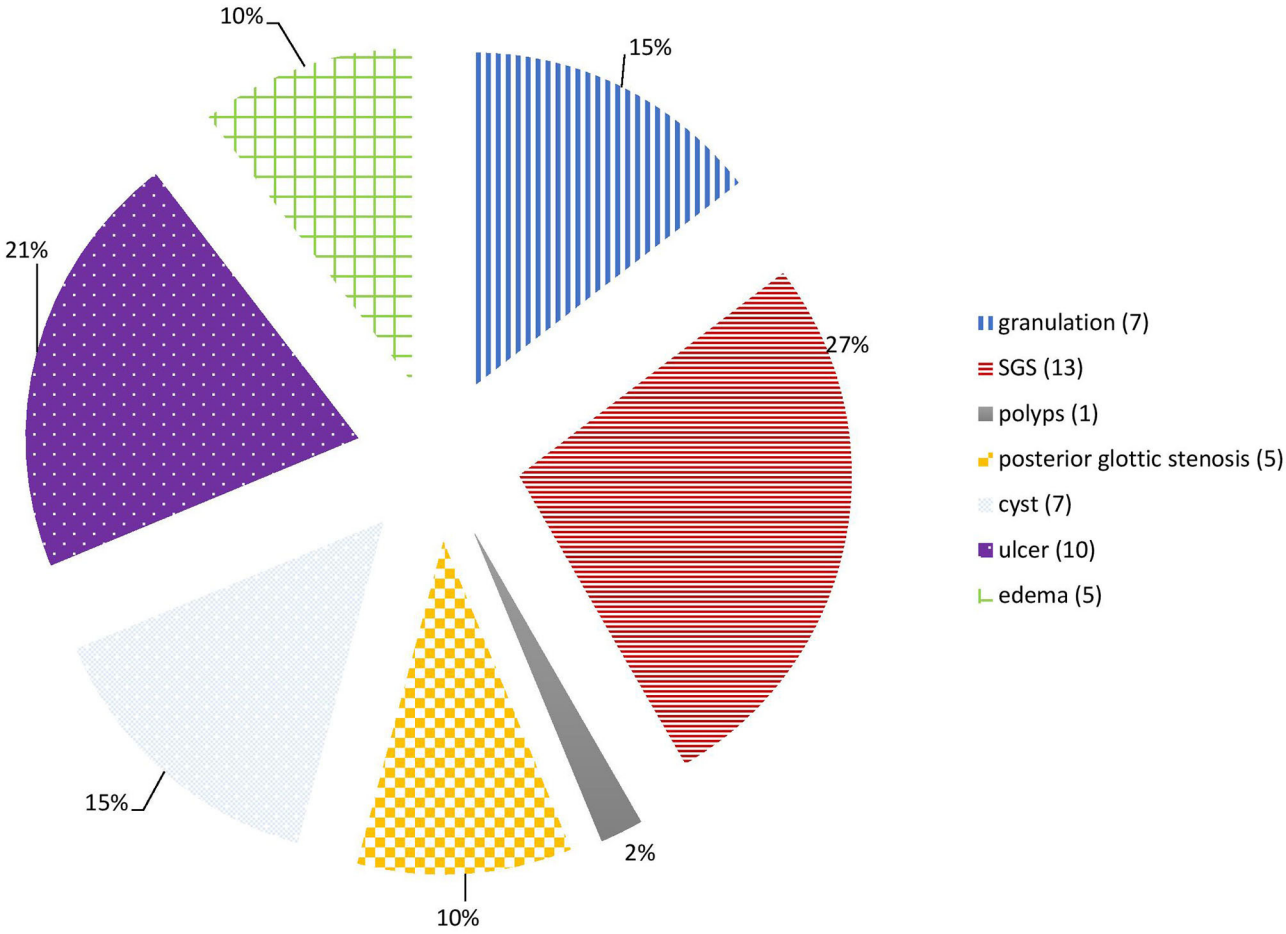
Intubation related lesions.

**Figure 2:**
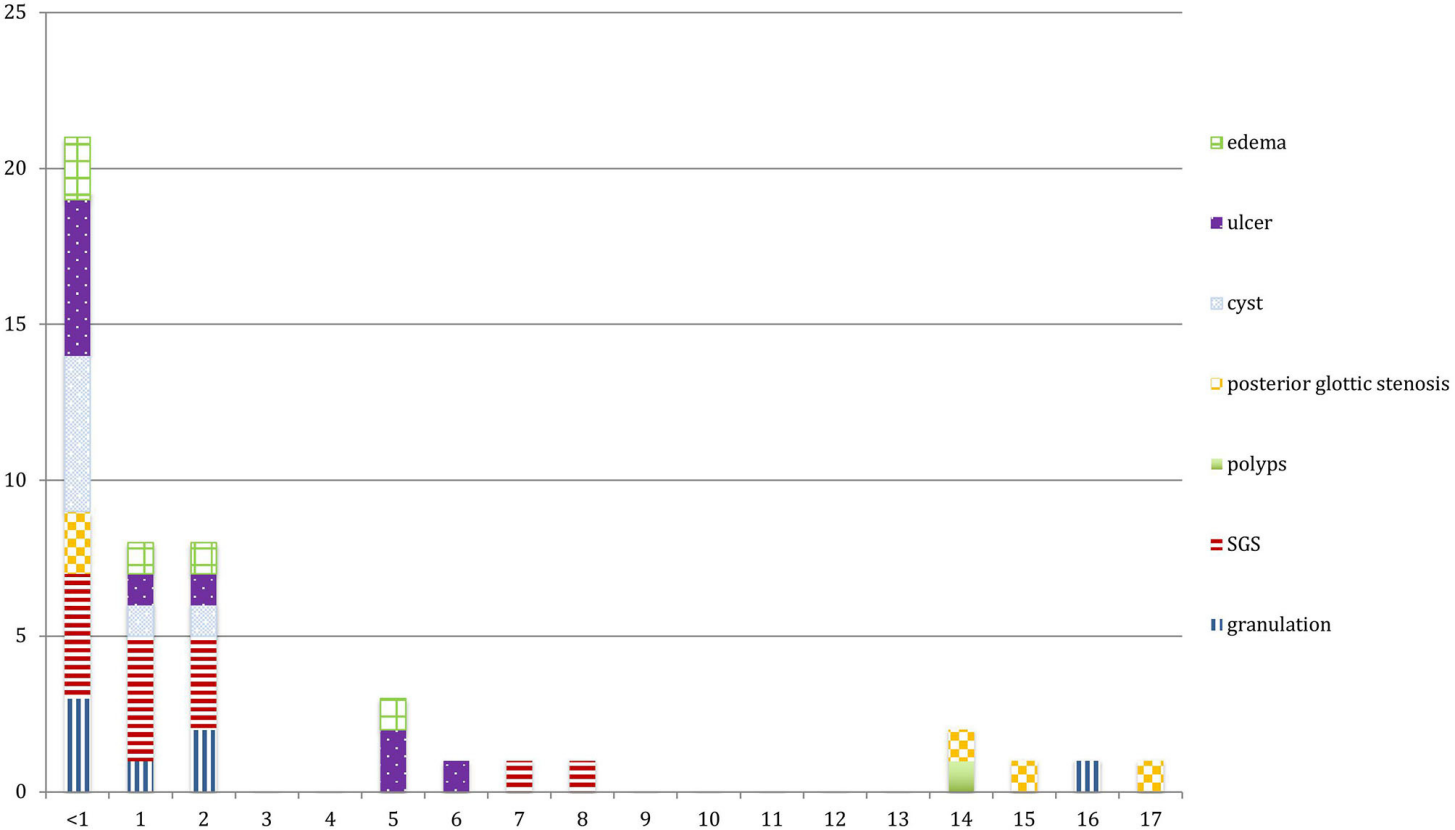
Lesions depending on the age. SGS, Subglottic stenosis.

The main LIRL were subglottic stenosis (12 patients, 31%), arytenoid ulcers (10 patients, 26%), granulations (7 patients, 18%), retention cysts (7 patients, 18%), posterior glottic stenosis (5 patients, 13%) and vocal cords edema (5 patients, 13%). Most of these lesions affected the subglottic region (27 patients, 69%), and the glottis was involved in 17 patients (46%). Posterior commissure was involved in 21 patients (54%), and bilateral vocal cords in 31 patients (79%) (Figures 1, 2; Table 1).

Symptoms suggesting the diagnosis of IRL included persistent stridor after extubation (20 patients, 51%), dyspnea (15 patients, 38%), dysphonia (7 patients, 18%), and aphonia (1 patient, 2.5%). One patient (2.5%) was asymptomatic and the lesion was found fortuitously. Almost half the cases (19 patients, 49%) were symptomatic in the first 24 h after extubation.

Favorable lesions (FL) occurred in 15 patients (38%), and unfavorable lesions (UFL) in 24 patients (62%). UFL were associated with prolonged intubation (> 1 week) (Average = 23 days, CI 95% [15.4–30.8], *p* < 0.05; relative risk 1.66; odds ratio 3.2).

We did not find any correlation between the lesion types and the indication and mode of intubation.

Two patients (5%, had grade II incipient SGS with 60 and 70% obstruction respectively) underwent open surgery to avoid a tracheotomy (both had single stage anterior and posterior cricoid splits with rib cartilage laryngoplasty). One patient received tracheotomy and simultaneous endoscopic LT Mold insertion. Currently, all these three patients and those who were treated endoscopically (*n* = 31; total = 34, 87%) report no breathing or severe voice complaints. Five patients (13%) with substantial comorbidities (2- prematurity; 2- bilateral vocal cord palsy; 1- astrocytoma) continue to have tracheostomy (Table 2).

**Table 2:** Management of LIRL.

**Lesion**	**Prevalence (*n*)**	**Management (*n*)**
Granulations	7 (18%)	Excision with biopsy forceps (6) - CO2 Laser vaporization (1)
Subglottic stenosis	12 (31%)	Endoscopic management - Balloon dilation (4) - Cold steel granulation excision + MMC application (6) Open surgery (2) - Both patients had incipient grade II stenosis(Cotton-Myer)
Polyps	1 (2.5%)	Cold steel excision + MMC application (1)
Posterior glottic stenosis	5 (12.8%)	Endoscopic management: - Stenotic band section with CO2 laser (2) - Cold steel granulation excision + MMC (3)
Cyst	7 (18%)	Marsupialisation with CO2 laser (7)
Arytenoid Ulcer(s)	10 (26%)	No treatment (8) Surgical debridement (1) Transtubation with a smaller tube (1)
Œdème	5 (12.8%)	No treatment (5)

*MMC, Mitomycin C*.

## Discussion

### Patients' Characteristics

As in several studies [6], LIRL in this study affected every age group, with the majority of patients being under 2 years.

Typically, the main indications for intubation at or immediately after birth are prior to surgical correction of a congenital defect, prematurity and congenital laryngeal malformations. Children are more vulnerable to develop LIRL than adults (12) due to difference in their laryngeal anatomy (13). The infant larynx is located much higher in the neck and is more anteriorly placed. Being rostral in position, the thyroid cartilage is concealed behind the hyoid bone. The caudal portion of the thyroid cartilage and the anterior cricoid arch in a child are inwardly bent as compared to a straighter angle in adults. This axis makes the endotracheal tube (ETT) tip abut against the cartilages causing intubation trauma. Syndromic children with limitation in mouth opening, retrognathism and oro-cervical masses provide additional technical challenges. Hence, the intubation in neonates, infants and syndromic children needs experience and understanding of their challenging anatomy.

The mucosae of the supra- and subglottis are lax in infants and hence more prone to develop edema. Therefore, the ETT size must be correctly chosen in small larynges (as in Downs syndrome) to avoid inducing trauma and development of intubation lesions. The mucosae of the supra- and subglottis are lax in infants and hence more prone to develop edema. Therefore, the ETT size must be correctly chosen in small larynges (as in Downs syndrome) to avoid inducing trauma and development of intubation lesions. More than half the patients in our study were premature. It is crucial to use an age-appropriate size tube that can optimally ventilate the child. Unfortunately, because several patients were referred to us from other centers, we have no data concerning the tube size used in the first place. This could have given interesting information about an eventual relationship between the tube used, the patient's age and the severity of LIRL.

Histopathologic changes in the larynx have been noted in neonates intubated for periods as short as several minutes to <12 h (14). For these reasons, some authors recommend post-extubation fiberoptic laryngoscopy in the pediatric population (4), though we do not systematically follow this policy.

### Lesions' Characteristics

Since the ETT rests in the posterior part of the larynx, the sites most vulnerable to an intubation injury are the inter-arytenoid region, medial surface of the arytenoids and posterior one-third of the vocal cords, the cricoarytenoid joints, and the posterior cricoid cartilage. Animal models have helped us to understand the pathophysiology of the LIRLs development and this has allowed developing a grading scale for the degree of injury (2, 3, 15–18). Mucosal ischemia occurs when decubitus pressure caused by the ETT is greater than the capillary pressure. These effects begin with non-specific mucosal alterations like irritation, hyperemia and edema (Grade 1). It then progresses to develop ulceration or necrosis with involvement of subepithelial layers (Grade 2), and later deep ulceration or necrosis reaching the cartilage (Grade 3). Within 48 h, granulation tissue develop and continue to proliferate the following days, sometimes encircling the surface of the tube on either side. The granulation tissue may act as a catalyst for chronic inflammation and perpetuate a vicious cycle of poor wound healing (18). Occasionally, *tongues* of granulation tissue developed in the posterior commissure become adherent across the midline and form a fibrous band between the arytenoid cartilages (inter-arytenoid adhesion) and limits vocal cord abduction. Florid granulations filling the posterior larynx may subsequently scar and create posterior glottic stenosis. Similarly, contraction of concentric scar tissue in the subglottic region evolves into subglottic stenosis (17). Scar tissue can spread into either one or both cricoarytenoid joints and cause vocal cord(s) fixity.

All these pathophysiological processes are facilitated by the following risk factors (14, 17):

- Patient related: prematurity, congenital cricoid cartilage malformation, systemic disease and immunodeficiency. In particular, hypoxia (cardiorespiratory causes and shock) has been shown to increase the severity of injury (2), suggesting that patients with acute or chronic hypoxia might be at greater risk.

Following extubation in a patient with LIRL, we systematically perform an endoscopy after 7–10 days for 2 reasons: 1] to look for the healing and evolution of the lesions and 2] note the laryngeal morphological causes (cricoid malformations) that could have increased the propensity to trauma and were not visualized during the first intubation.

- Intubation technique related: tube selection, mode, duration of intubation.
- Post-intubation conditions: inadequate sedation with agitation, excessive stasis of laryngeal secretions, bacterial contamination, gastroesophageal reflux, presence of a large and hard nasogastric tube that squeeze the post cricoid and posterior commissure regions against the endotracheal tube thus causing pressure necrosis and ulceration.

Our study included intubation lesions of different types and severity. We found a correlation between stenosing (unfavorable) lesions and prolonged intubation which is in agreement with the previous publications (5, 14).

### Acute and Long-Term Management of IRLs

**Figure 3:**
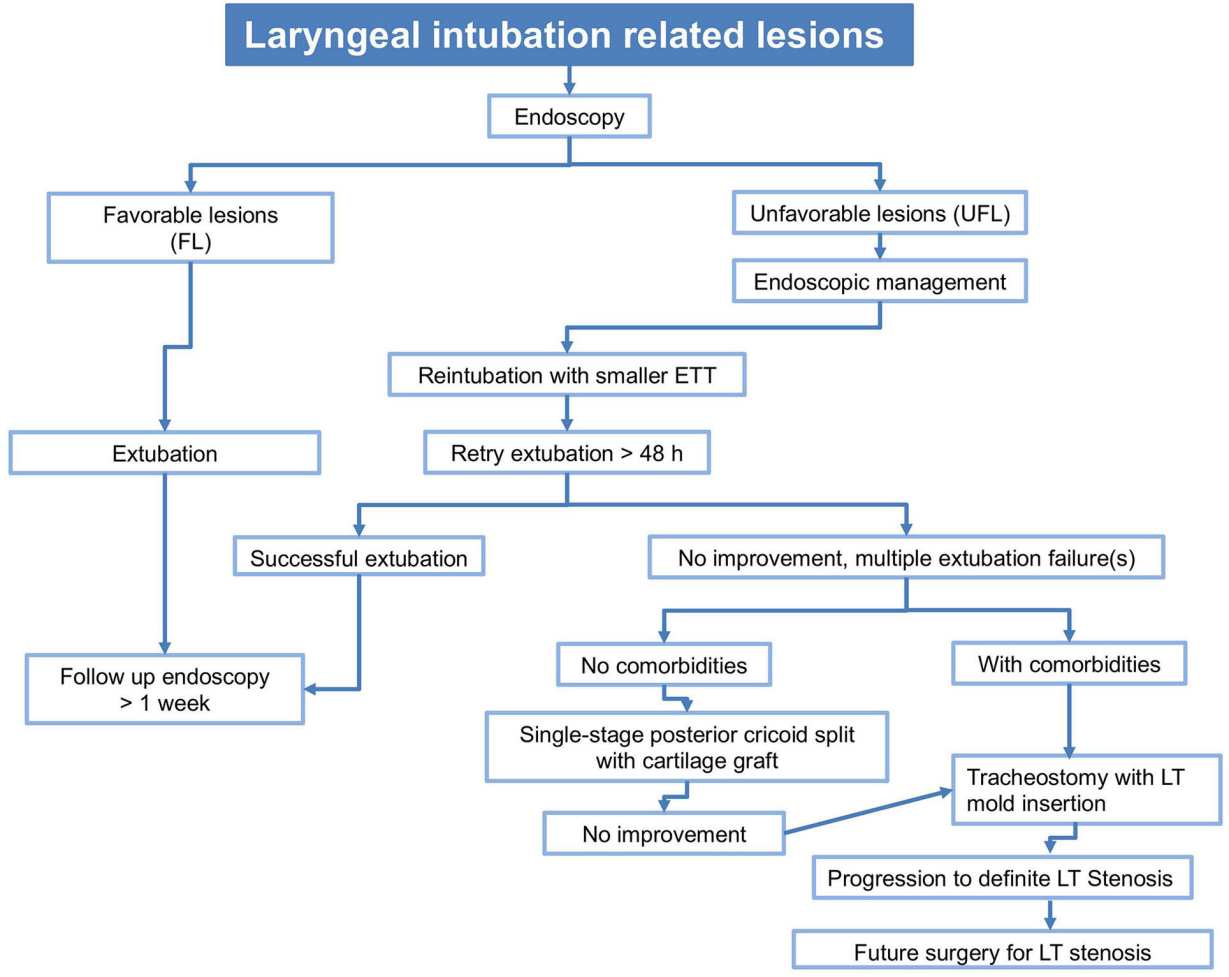
Algorithm to manage LIRL. FL: erythema, mild edema, small, non-obstructing subglottic retention cyst(s). UFL: moderate-severe edema with mucosal ulceration,posterior glottic and circumferential subglottic granulations, non-obstructing subglottic retention cyst(s),severe bilateral arytenoids and circumferential cricoid cartilage exposure, severe cuff/tip of the tube lesions. Comorbidities: cardiopulmonary, neurologic, syndromic.

Management of acute IRLs is critical to prevent them from evolving into more serious airway obstruction. Literature concerning management of IRLs is scanty, with no universal consensus and currently is based on the surgeons' experience and the clinical impression one gets during the first and subsequent endoscopies. We follow an algorithm to manage LIRLs (Figure 3), though its validation needs larger number of patients and additional studies.

Patients with obstructive retention cyst(s) were treated by CO2 laser marsupialization. In cases of bilateral severe subglottic cysts, we prefer treating them in two stages to avoid creating circumferential raw area that can develop into subglottic stenosis. Laser use in an infantile larynx has a learning curve and appropriate training is a prerequisite to avoid iatrogenic-induced collateral damage.

For incipient subglottic stenosis, we prefer using cold-steel instruments to remove simple or florid granulation tissue. Use of CO2 laser is contraindicated in such cases as it is likely to ccarbonize highly vascularized granulation tissue, thereby generating heat diffusion into the surrounding tissues and subsequent scarring (16). Apart from this thermal-induced collateral damage, fiber driven lasers can be cumbersome to use in neonates and small infants due to their small airways and possible anesthesia challenges. Aggressive removal of granulation tissue is important, though its over-doing should be avoided to avoid iatrogenically induced stenosis. We used Mitomicin C (MMC) in cases with florid granulations and avoided its use on an exposed cartilage, as this delays reepithelization process and promotes necrosis by long-standing exposure of the cartilage in the subglottic lumen (16). MMC has been found to modulate wound healing response by inhibiting both neoangiogenesis and fibroblast proliferation. It must be used judiciously because—apart from being carcinogenic, it may cause excess fibrin formation and hence airway obstruction (16, 19).

Thin, cicatricial bands and circumferential stenosis can be incised using cold steel instruments or CO2 laser after prior injection of triamcinolone acetonide into the stenosis and subsequent balloon dilation of the stenotic airway. Balloon dilation will crush granulation tissue that can then be removed using microlaryngeal forceps. However, we did not find balloon dilation of edematous mucosa useful as has been mentioned by Schweiger et al. (9).

Adrenaline is used for hemostasis and topical gentamicin-betamethasone ointment is applied. The patient is then re-intubated with one size smaller tube and the extubation is attempted 48 h later. In case of an extubation failure due to severe incessant intubation lesions and if tracheotomy is performed, we feel it essential to treat the proximal subglottic stenosis (SGS) by endoscopically inserting an LT mold. This allows healing of the larynx with vocal cords in abduction and thus maintains an appropriately sized glottis. A prosthesis will also prevent laryngotracheal affection secondary to gastroesophageal reflux, and allow better healing.

Conservative treatment can be sufficient in most cases of LIRL. Medical treatment should include systemic corticosteroids, adrenaline and steroid aerosols, proton pump inhibitors and antibiotics (9). We entirely agree with Wei et al. (15) that meticulously performed endoscopic approach as primary treatment modality is critical in reducing the worsening of airway stenosis due to LIRL.

In selected cases of severe incipient stenosis, we prefer attempting endoscopic and expansion airway procedures to avoid a tracheotomy as opposed to some centers (20) who recommend it upfront in similar cases. The site of performing the tracheotomy in case of a failed extubation is very important. In anticipation of future treatment for laryngotracheal stenosis (LTS), it must be performed either between the cricoid and the first tracheal ring or between the sixth and seventh rings. This allows performing either a single stage procedure (meaning concomitant stenosis treatment and removal of the stoma) or treatment in two stages (stenosis treatment in the first stage and decannulation planned in the second).

### Prevention

Apart from LIRL treatment, particular consideration should be given to their prevention. Selection of the ETT size according to the child's age is well-established (20). Usually, a rough estimation can be made by comparing the outer diameter of the tube with the width of the child's little finger, or by using the formula ([age in years + 16] and then divide by 4).

Severity of laryngeal injuries induced by the ETT is directly related to the cuff pressure. When this pressure exceeds mucosal perfusion pressure (25 mmHg), it affects the mucosal vascularity and traumatizes the perichondrium—thus leading to ulceration, bacterial contamination and chondritis (21). The choice of ETT with or without cuff in children is still widely debated, although some authors recommend an uncuffed tube in children under 7 years (17). Weiss et al. showed that cuffed tracheal tubes in small children (<5 years) provide a reliably sealed airway at cuff pressures <20 cmH2O, and additionally does not increase the risk for post-extubation stridor in comparison with uncuffed ETTs (22).

Inadequate sedation risks accidental extubation, agitation amongst intubated children and provokes constant friction between the tube and the vocal cords. This mechanical trauma along with mucosal damage evokes dangerous laryngotracheal lesions (21).

Additional measures to prevent or reduce the incidence of LIRL include optimal treatment of gastroesophageal reflux and adequate nursing (17).

There are several drawbacks in our study. Primarily it is monocentric, retrospective, observational in nature, and has heterogeneous patient groups. LIRLs in the pediatric age group are rare and has serious consequences. We hope this commentary will initiate an interest among researchers to conduct prospective, large cohort and multi-centric studies aimed at reducing this iatrogenic-induced morbidity.

## Conclusion

LIRL management can be challenging and stressful in neonates, infants, and small children. Timely and adequate treatment can avoid extreme morbidity in these patients. Long intubation duration and associated comorbidities are critical factors deciding the severity of these lesions. Establishing a treatment strategy and having prevention measures are important.

## Data Availability Statement

The data analyzed in this study is subject to the following licenses/restrictions: Data were anonymized and available only to the authors. Requests to access these datasets should be directed to Laurence Pincet, laurencepincet@gmail.com.

## Author Contributions

KL and LP conceptualized and designed the study, drafted the initial manuscript, reviewed, and revised the manuscript. KS conceptualized and designed the study, coordinated and supervised data collection, and critically reviewed the manuscript for important intellectual content. All authors approved the final manuscript as submitted and agree to be accountable for all aspects of the work.

## Conflict of Interest

The authors declare that the research was conducted in the absence of any commercial or financial relationships that could be construed as a potential conflict of interest.
